# Signaling events induced by lipopolysaccharide-activated Toll in response to bacterial infection in shrimp

**DOI:** 10.3389/fimmu.2023.1119879

**Published:** 2023-02-03

**Authors:** Sheng Wang, Haoyang Li, Qinyao Li, Bin Yin, Sedong Li, Jianguo He, Chaozheng Li

**Affiliations:** ^1^ State Key Laboratory of Biocontrol/Southern Marine Science and Engineering Guangdong Laboratory (Zhuhai), School of Marine Sciences, Sun Yat-sen University, Guangzhou, China; ^2^ Guangdong Provincial Key Laboratory of Marine Resources and Coastal Engineering/Guangdong Provincial Key Laboratory for Aquatic Economic Animals, Guangzhou, China; ^3^ China-Association of Southeast Asian Nations (ASEAN) Belt and Road Joint Laboratory on Marine Aquaculture Technology, Guangzhou, China; ^4^ Guangdong Evergreen Feed Industry Co., Ltd, Zhanjiang, China; ^5^ Guangdong Laboratory for Lingnan Modern Agriculture, Maoming, China

**Keywords:** shrimp, Toll, TAK1/TAB2/TRAF6 complex, Vibrio parahaemolyticus, dorsal

## Abstract

Toll-like receptors (TLR) play a crucial role in the detection of microbial infections in vertebrates and invertebrates. Mammalian TLRs directly recognize a variety of structurally conserved microbial components. However, invertebrates such as Drosophila indirectly recognize microbial products by binding to the cytokine-like ligand Spätzle, which activates signaling cascades that are not completely understood. In this study, we investigated the signaling events triggered by Toll in response to lipopolysaccharide (LPS), a cell wall component of gram-negative bacteria, and *Vibrio parahaemolyticus* infection in the arthropod shrimp *Litopenaeus vannamei*. We found that five of the nine Tolls from *L. vannamei* bound to LPS and the RNAi of LvToll1, LvToll2, LvToll3, LvToll5, and LvToll9 weakened LvDorsal-L phosphorylation induced by *V. parahaemolyticus*. All nine Tolls combined with MyD88 *via* the TIR domain, thereby conferring signals to the tumor necrosis factor receptor-associated factor 6 (TRAF6)-transforming growth factor-β activated kinase 1 binding protein 2 (TAB2)-transforming growth factor-β activated kinase 1 (TAK1) complex. Further examination revealed that the LvTRAF6-LvTAB2-LvTAK1 complex contributes to Dorsal-L phosphorylation and nuclear translocation during *V. parahaemolyticus* infection. Overall, shrimp Toll1/2/3/5/9–TRAF6/TAB2/TAK1–Dorsal cascades protect the host from *V. parahaemolyticus* infection, which provides a better understanding of how the innate immune system recognizes and responds to bacterial infections in invertebrates.

## Highlights

1. Shrimp Tolls directly bind to lipopolysaccharide (LPS).

2. A new isoform of Dorsal (Dorsal-L) identified confers protection against bacterial infection.

3. The Tolls–TRAF6/TAB2/TAK1–Dorsal-L (NF-κB) antibacterial axis is identified in shrimp.

## 1 Introduction

The Toll-like receptor (TLRs) signaling pathway is a non-negligible pathway involved in resistance to microbial invasion (1). To date, 13 TLRs have been identified in mammals (2). Mammalian TLRs, such as those found in mice, directly recognize specific microbial products called pathogen-associated molecular patterns (PAMPs) as well as bacterial lipoproteins. The 13 TLRs identified in mammals include: TLR1, TLR2, and TLR6 (bacterial lipoproteins) (3); TLR3 (double-stranded RNA) (4); TLR4 (lipopolysaccharide, LPS) (5); TLR5, TLR11, and TLR12 (bacterial peptide flagellin) (6–8); TLR7 and TLR8 (single-stranded RNA) (9, 10); TLR9 (CpG motifs within DNA) (11); and TLR13 (bacterial 23S ribosomal RNA) (12). After sensing PAMPs, TLR-mediated MyD88-dependent or MyD88-independent signaling cascades are activated through the recruitment of Toll/IL-1 receptor (TIR) domain-containing adaptors, such as myeloid differentiation factor 88 (MyD88), TIR domain-containing adaptor protein (TIRAP), and TIR domain-containing adapter-inducing interferon-beta (TRIF) (13). MyD88 is the classical TIR domain-containing adaptor that links TLRs to IL-1R-associated kinases (IRAKs) (homologs of Tube and Pelle in invertebrates). Recruitment of IRAK proteins leads to the activation of NF-κB in a TRAF6-dependent manner (14). TRAF6 activates the downstream kinase TAK1 in complex with transforming growth factor-β activated kinase 1 binding protein 2 (TAB2) and transforming growth factor-β activated kinase 1 binding protein 3 (TAB3), thereby allowing TAK1 to activate the IkappaB kinase (IKK) complex, which catalyzes the phosphorylation of IκB proteins, resulting in the nuclear translocation of NF-κB (13, 15). NF-κB can bind to κB sites in the promoters of target genes such as pro-inflammatory factors and antimicrobial peptides (AMPs), which leads to their transcription (16–19).

The recognition and signal transfer mechanisms of the Toll pathway in invertebrates are different from those in vertebrates. The Toll pathway in Drosophila primarily responds to gram-positive bacteria and fungi but not to gram-negative bacteria (20). Moreover, Toll does not function as a pattern recognition receptor (PRR) (21). Immune factors secreted by peptidoglycan recognition proteins (pGRP-SA and pGRP-SD) and the gram-negative bacillary pneumonias (GNBP) family member, GNBP1, act as PRRs and initiate NF-κB by binding to fungal β-1, 3-glucan, or G^+^ bacterial peptidoglycan. The PAMP-PRR combination triggers serine protease cascade activation, hydrolyzing the ligand of the Toll receptor pro-Spätzle to Spätzle, which combines with the Toll receptor, thereby activating the signaling pathway. Once activated, MyD88, Tube, and Pelle form a complex, therefore leading to the degradation of Cactus, allowing Dorsal and Dorsal immunity factor (DIF) nuclear translocation and AMPs expression. However, the TRAF6 homolog, dTRAF2, is not essential in response to bacterial infection (22), and the mechanism of signal transfer in the Toll pathway of invertebrates remains unclear.

Although shrimp *L. vannamei* is an invertebrate, there are many indications that the shrimp Toll differs from Drosophila in recognition and signal transduction. Unlike the Toll pathway in Drosophila, the shrimp Toll pathway responds to both *Staphylococcus aureus* (gram-positive bacteria) and *Vibrio parahaemolyticus* (gram-negative bacteria) infection (23). Furthermore, components of the shrimp Toll pathway induce a broad spectrum of AMPs, which can remove pathogens such as gram-positive and gram-negative bacteria (23). Although it has been established that the shrimp Toll pathway responds to bacterial infection and has a role in regulating AMPs expression, the mechanism responsible for the bacterial initiation of the *L. vannamei* Toll pathway and activation of the immune response is unknown. In this study, we found that LvToll1/2/3/5/9 specifically recognized LPS and stimulated the translocation of LvDorsal-L from the cytoplasm to the nucleus *via* the LvTRAF6-LvTAB2-LvTAK1 complex and activated LvCrustin1 transcription, which plays an antibacterial role during *V. parahaemolyticus* infection. The shrimp Toll-mediated anti-*V. parahaemolyticus* signaling pathway provides a deeper understanding of invertebrate pathogen recognition and signaling mechanisms as well as the evolution of innate immunity in both vertebrates and invertebrates.

## 2 Materials and methods

### 2.1 Animals

HAID Group provided healthy *L. vannamei* with a body weight of about 5 g each. Prior to any experiments, the shrimp were acclimated in aerated artificial seawater (25 salinity) for two days. A commercial shrimp diet was fed to the shrimp on a daily basis (HAID Group).

### 2.2 Plasmids construction

The open reading frames (ORFs) of *L. vannamei* Dorsal-L and Dorsal-S were amplified and cloned into pAc5.1-3×HA vector (24) to express the HA-tagged Dorsal-L protein and Dorsal-S proteins, respectively. Extracellular (ec) domain of each Toll (Toll1/2/3/4/5/6/7/8/9-ec), and the cytoplasmic TIR domain of each Toll (TIR1/2/3/4/5/6/7/8/9) were constructed into pAc5.1-3×HA vector to express HA-tagged proteins. The full length of TRAF6 (1–594), and a series of truncated mutants of TRAF6, including TRAF6 (1-100), TRAF6 (101-225), TRAF6 (226-410) and TRAF6 (411-594), were all inserted into pAc5.1-3×HA vector. The full length of TAB2 (1-608) was constructed into pAc5.1-3×FLAG vector (25), while different truncated mutants of TAB2, including TAB2 (1-60), TAB2 (61-320), TAB2 (321-410), TAB2 (411-500), TAB2 (501-530) and TAB2(531-608), were constructed into pAc5.1-GFP vector (26). Besides, two truncated mutants of TAK1, including TAK1 (1-630) and TAK1 (631-758) were also constructed into pAc5.1-GFP vector. All the primers used for vector construction were shown in **Supplementary Table 1**.

Protein expression plasmids, including TAK1-GFP, TAK1-V5, TAB2-GFP, MyD88-GFP, Tube-GFP, and Pelle-GFP, and the reporter gene vectors, including DmDpt-pGL3, DmDrs-pGL3, DmAttA-pGL3, Pm411-pGL3, Pm536-pGL3, LvCrustin1-pGL3, 4×NF-κB-pGL3, and pRL-TK, were obtained from our previous studies (27–29).

### 2.3 Pull-down, co-immunoprecipitation and western blotting

To explore the potential interaction between LPS and each Toll, the plasmids expressing the extracellular (ec) domain of each Toll (Toll1/2/3/4/5/6/7/8/9-ec-HA), or pAc5.1A-3×HA (as a control), were transfected in Drosophila S2 cell line, respectively. Forty-eight hours post- transfection, cells were lysed in IP lysis buffer (Pierce, cat. no. 87788) with Halt Protease Inhibitor Cocktail (Thermo Fisher Scientific, cat. no. 87786). The supernatants were incubated with 10 μg LPS-EB Biotin (*In vivo*Gen, cat. no. tlrl-lpsbiot) at 4 °C for half an hour, followed by incubation with Monoclonal Anti-biotin agarose antibody produced in mouse (Merck, cat. no. A1559-5ML) at 4 °C for 2 hours.

An *in vitro* co-immunoprecipitation (Co-IP) assay was performed to detect the interaction between different pair of proteins. In brief, 48 hours after transfection, Drosophila S2 cells were lysed in IP lysis buffer (Pierce, cat. no. 87788) with Halt Protease Inhibitor Cocktail (Thermo Fisher Scientific, cat. no. 87786). The supernatants were incubated with 30 µl of Monoclonal Anti-HA Agarose antibody produced in mouse (Merck, cat. no. A2095) or aAnti-GFP mAb Agarose (MBL International Corporation, cat. no. D153-8) or an Anti-V5 Agarose Affinity Gel antibody produced in micouse (Merck, cat. no. A7345) or ANTI-FLAG M2 Affinity Gel (Merck, cat. no. A2220-25ML) at 4 °C for two hours.

The agarose (from pull-down or co-immunoprecipitation) was washed five times with PBS before being subjected to SDS-PAGE and the western blot assays. In addition, 5% of total cell lysis was used tested as an input control. Membranes were developed with the Omni-ECL enhanced Pico Light Chemiluminescence Kit (EpiZyme, cat. no. SQ101L), and chemiluminescence was detected using the 5200 Chemiluminescence imaging System (Tanon).

### 2.4 Antibodies

The primary antibodies used in this study included rabbit anti-GFP N-terminal antibody produced in rabbit (Sigma-Aldrich, cat. no. G1544-100UL), Anti-V5 Epitope Tag Antibody (Merck, cat. no. AB3792), rabbit anti-HA antibody produced in rabbit (Merck, cat. no. H6908-100UL), rabbit anti-FLAG antibody produced in rabbit (Merck, cat. no. F7425), rabbit anti-p-LvDorsal (Genecreate), rabbit anti-LvDorsal (Genecreate), and mouse anti-actin clone C4 antibody (Merc, cat. no. MAB1501) and Monoclonal anti-β-Actin antibody produced in mouse (Merck, cat. no. A2228). The secondary antibodies used were anti-mouse IgG horseradish peroxidase (HRP-conjugate) (Promega, cat. no. W4021), anti-rabbit IgG HRP-conjugate (Promega, cat. no. W4011), anti-rabbit IgG (H+L) F (ab’)2 fragment Alexa Fluor 488 Conjugate (CST, cat. no. 4412S), and anti-mouse IgG (H+L), F (ab’)2 Fragment Alexa Fluor 594 Conjugate (CST, cat. no. 8890S).

### 2.5 Targeted gene silencing using dsRNA-mediated RNAi

The dsRNA-Dorsal-L, dsRNA-Dorsal-S, dsRNA-Dorsal-B (targeting both Dorsal-L and Dorsal-S), and dsRNA-GFP (as a control) were synthesized by *in vitro* transcription with T7 RiboMAX Express RNAi System kit (Promega, cat. no. P1700) (**Supplementary Table 1**). The dsRNAs targeting Toll1/2/3/4/5/6/7/8/9, TAK1, and TRAF6 were obtained from our previous study (30, 31). Each shrimp was given an intraperitoneal injection of dsRNAs (2 μg/g shrimp in 50 µl of PBS) or an equivalent volume of PBS for gene silencing. Hemocytes were collected from shrimp at 48 hours after the dsRNA injection to evaluate the efficacy of RNAi (**Supplementary Table 1**).

### 2.6 Shrimp challenge, sampling, and survival experiments

*V. parahaemolyticus* (MCCC: 1A10122), which was isolated from the hepatopancreas of diseased shrimp *L. vannamei*, was cultured in Luria broth (LB) medium overnight at 37°C. The microbial colony-forming units (CFUs) per milliliter of cultured *V. parahaemolyticus* were counted on LB agar plates. Shrimp were injected with 50 µl *V. parahaemolyticus* (approximately 1 × 10^5^ CFU). Six hours later, the hemocytes of three shrimp were sampled for detecting the phosphorylation levels of Dorsal.

Each shrimp was injected with 10 µg of dsRNA. And shrimp was received a second injection with PBS or 50 µl *V. parahaemolyticus* (approximately 1 × 10^5^ CFU) after 48 hours. The number of died shrimp was recorded every 4 h for five days.

Parallel experiments were also performed to sample shrimp tissues for further detection. Hemocytes were collected from at least three shrimp at 48 hours post dsRNA injection (for qPCR to detect the efficiency of RNAi), 6 hours post *V. parahaemolyticus* injection (for Western blotting and Immunofluorescence), and 48 hours post *V. parahaemolyticus* injection (for qPCR to detect the transcription level of LvCrustin1). The control C4 blots were derived from the same samples of the p-Dorsal blot.

### 2.7 Quantitative PCR (qPCR)

QPCR were used to assess the transcription levels of target genes. The cDNA templates for the tissue distribution assay and challenge experiments were obtained according to a previous study (28). The expression levels of Dorsal-S, Dorsal-L, Toll1-9, and Crustin1 were determined and calculated by qPCR and Livak (2^-△△CT^) method (32) after normalization to *L. vannamei* elongation factor 1 alpha (EF1-α). All samples were tested in triplicate. Primer sequences were listed in **Supplementary Table 1**.

### 2.8 Dual luciferase reporter assay

The Dual-luciferase reporter assays were done as our previous described (24) and measured with Dual-Glo Luciferase Assay System kit (Promega, cat. No. E2940). Western blotting was used to examine 50% of cell lysis as inputs.

### 2.9 Immunofluorescence and confocal laser scanning microscopy

The hemocytes from dsGFP- injected shrimp, dsTAK1- injected shrimp or dsTRAF6- injected shrimp at 6 hours post- *V. parahaemolyticus* infection were collected for immunofluorescence with rabbit anti-LvDorsal and mouse anti-β-Actin antibody.

### 2.10 Statistical analysis

The data are all displayed as mean ± SD. To compare groups of numerical data, the Student’s *t*- test was applied. Data was statistically analyzed for survival rates using Kaplan– ± Meier plot (log-rank χ^2^ test). Statistical significance was defined as * *p* < 0.05, and ** *p* < 0.01.

## 3 Results

### 3.1 Shrimp Toll1/2/3/5/9 interacted with LPS

**Figure 1:**
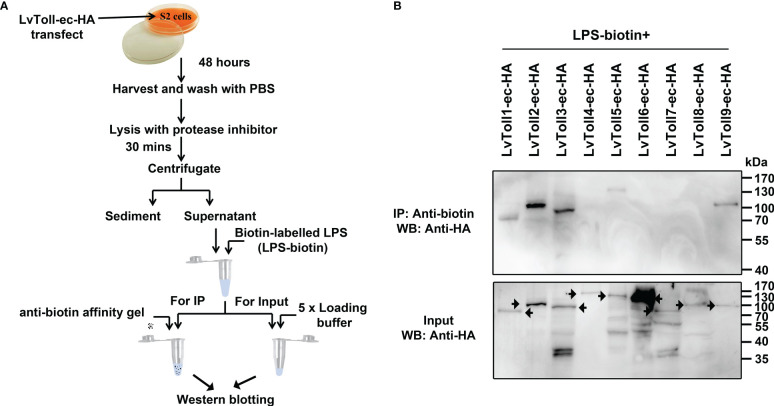
Screening a total of nine LvTolls that recognize LPS. **(A)** The flow chart of the *in vitro* LPS-LvTolls interaction experiments. The extracellular domain of LvTolls was individually over-expressed in S2 cells. Forty-eight hours after transfection, cells were harvested and lysed. The supernatant of cell lysis was incubated with biotin labeled LPS, which was further incubated with the anti-biotin agarose. **(B)** The interaction between LPS and LvToll-ec. The extracellular domain of Toll easily presents heterozonal bands. The black arrow stands for the band that corresponds to the LvToll-ec.

Mammalian TLR receptors function as PRRs that directly sense microbial components (33). To date, nine tolls have been identified in the shrimp, *L. vannamei* (31). To explore whether any of the nine Tolls could interact with LPS, pull-down assays were performed in Drosophila S2 cells (**Figure 1A**). The extracellular parts of Toll1, Toll2, Toll3, Toll5, and Toll9 were found to bind to LPS (**Figure 1B**), implying that shrimp Tolls which recognize LPS may have a pattern similar to mammalian TLRs that detect pathogenic PAMPs.

### 3.2 Shrimp Tolls combined with MyD88 lead to Tube-Pelle-TRAF6 cascade

**Figure 2:**
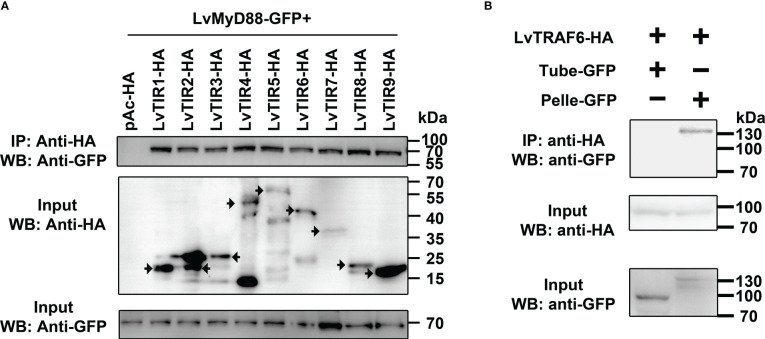
LvTolls interacted with LvMyD88 and conferred the signal *via* Tube-Pelle-TRAF6 cascade. **(A)** Co-IP assay showed that the TIR domain of all LvTolls interacted with LvMyD88. The black arrow stands for the band that the corresponds to the full length TIR domain. **(B)** Co-IP assay showed that LvTRAF6 could be co-precipitated by LvPelle but not LvTube.

MyD88 acts as an adaptor protein linking various Tolls/TLRs and downstream signal transduction proteins in the Toll/TLR pathways within mammals and Drosophila (31). Whether or not MyD88 can act as a downstream signaling mediator of the Toll pathway in shrimp has not yet been revealed. All nine Tolls and MyD88 contain one TIR domain. Thus, we explored the potential interaction between Toll and MyD88 using co-immunoprecipitation assays. We observed that GFP-tagged MyD88 was immunoprecipitated with each TIR domain of the nine Tolls (HA tag) with anti-HA antibody agarose affinity gels (**Figure 2A**). In addition, according to our previous studies, shrimp Tube (IRAK4 ortholog) acts as an adaptor protein to link MyD88 and Pelle (IRAK1 ortholog) to form a functional complex, MyD88-Tube-Pelle, that regulates Toll signaling (27). However, the downstream events of Pelle in shrimp and other invertebrates are still unclear. We inferred that shrimp TRAF6 might play a role in mediating signal transduction by interacting with Tube and Pelle. To address this, shrimp TRAF6 was co-expressed with Pelle or Tube, and we observed that HA-tagged TRAF6 interacted with GFP-tagged Pelle but not with the GFP-tagged Tube (**Figure 2B**). These findings suggest that, similar to TLR signaling in mammals, shrimp Tolls that sense LPS may initiate a signaling cascade involving the MyD88-Tube (IRAK4)-Pelle (IRAK1)-TRAF6 complex.

### 3.3 Identification of the TRAF6-TAB2-TAK1 complex in shrimp

**Figure 3:**
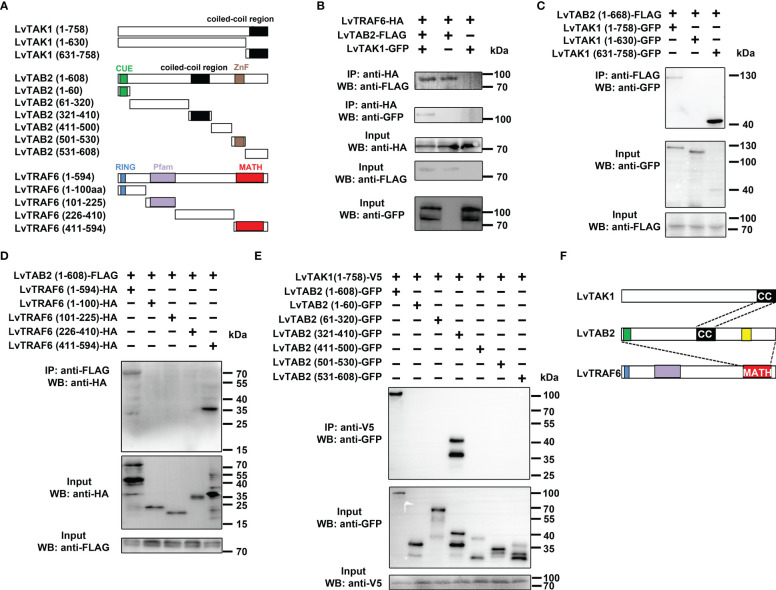
LvTRAF6, LvTAB2 and LvTAK1 could form a TRAF6-TAB2-TAK1 complex. **(A)** LvTAB2, LvTAK1, and LvTRAF6 interaction **(B)** Schematic representations of LvTAK1, LvTAB2, and LvTRAF6 segments. **(C)** Interaction of LvTAB2 and LvTAK1 segments **(D)** Interaction of LvTAB2 and LvTRAF6 segments **(E)** Interaction of LvTAK1 and LvTAB2 segments **(F)** Schematic representations of the LvTRAF6-LvTAB2-LvTAK1 complex.

Since mammalian TRAF6 can form a complex with TAB2-TAK1 to activate NF-κB, we reasoned that shrimp TRAF6 could be involved in the formation of the TRAF6-TAB2-TAK1 complex. *In vitro* co-immunoprecipitation assays were performed to confirm the presence of the shrimp TRAF6-TAB2-TAK1 complex, and the interaction domains were determined using a series of truncated mutants (**Figure 3A**). We demonstrated that TRAF6 could only interact with TAK1 in the presence of TAB2, while TAB2 could bind to both TRAF6 and TAK1 directly (**Figure 3B**), suggesting that TAB2 functions as an adaptor linking TRAF6 and TAK1. The results also showed that the full-length TAB2 (1-608) was immunoprecipitated with TAK1 (631-758), which contained the coiled-coil region (**Figure 3C**). The C-terminal MATH domain of TRAF6 interacted with TAB2 (**Figure 3D**). Interestingly, although the coiled-coil region of TAB2 (321-410) was vital for its interaction with TAK1 (**Figure 3E**), the full-length TAB2 was required for its interaction with TRAF6 (**Supplementary Figure 1**). In particular, TAB2 interacted with TAK1 through a combination of their coiled-coil regions, while the entire length of TAB2 was required to form a certain conformation to interact with the C-terminus of TRAF6 (MATH domain) (**Figure 3F**). Overall, our results indicate that shrimp TAB2 links TRAF6 to TAK1.

### 3.4 Dorsal-L was the dominant Dorsal isoform to induce antimicrobial peptides (AMPs)

**Figure 4:**
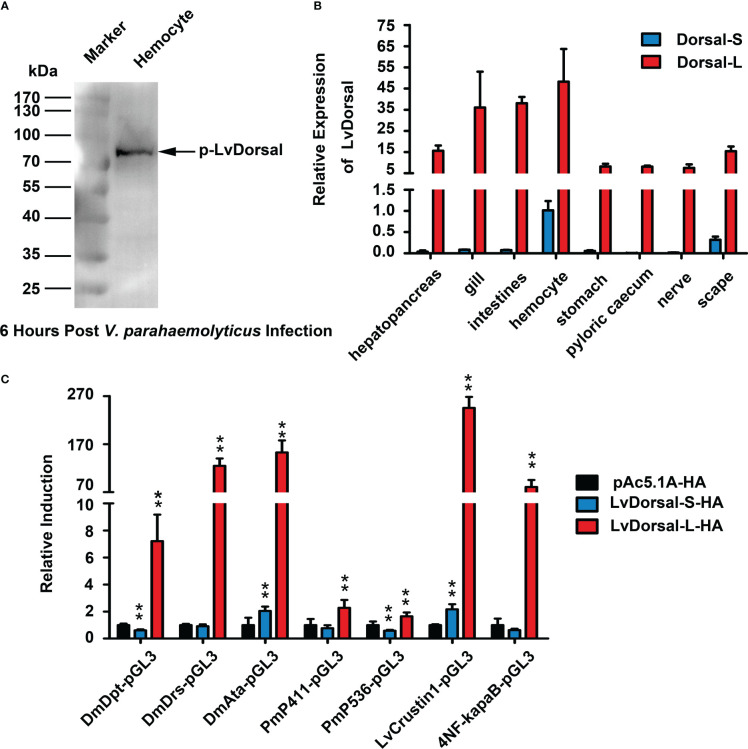
Characterization of LvDorsal-L. **(A)** LvDorsal phosphorylation in hemocytes of *V. parahaemolyticus*-infected shrimp. **(B)** LvDorsal-S and LvDorsal-L tissue distributions in healthy *L. vannamei*. **(C)** The effects of LvDorsals on the promoter activities of AMP genes and the NF-κB binding site. All the data **(B, C)** were provided as the means ± SD of at least three assays, and analyzed statistically by student’s T test (**: *p* < 0.01).

Dorsal is the NF-κB transcription factor that acts downstream of the Toll pathway in both shrimp and Drosophila (31, 34). Dorsal phosphorylation may reflect the activation of the Toll pathway (31). To determine whether LvDorsal responded to *V. parahaemolyticus* infection, the hemocytes from infected shrimp were detected by western blotting using the p-LvDorsal antibody based on phosphorylation Ser342 in the ‘VQLLRPSDKST’ peptide. Interestingly, the band appeared to be over 70 kDa (**Figure 4A**), whereas the LvDorsal band was only ~44 kDa. As a result, we believe in addition to the previous LvDorsal, another Dorsal of *L. vannamei* may exist. A new LvDorsal (ROT84343.1) was discovered after blasting in National Center for Biotechnology Information (NCBI), and the sequence was identified using PCR. Because ROT84343.1 was substantially larger than the previous LvDorsal (ACZ98167.1), we termed ROT84343.1 LvDorsal-L and ACZ98167.1 LvDorsal-S. The LvDorsal-L sequence is shown in **Supplementary Figure 2A**. Similar to Dorsal in other species, architecture prediction showed that LvDorsal-L possessed a Rel-homology domain (RHD) DNA-binding domain, followed by an RHD dimer domain (**Supplemental Figure 2B**). Multiple sequence alignment revealed that the full-length amino acid sequence of LvDorsal-S was almost fully consistent with the N-terminal of LvDorsal-L, with only three aa missing in the N-terminal and 10 aa differences in the C-terminal (**Supplementary Figure 3**).

qPCR was used to determine the tissue distribution of LvDorsal-L and LvDorsal-S. Both LvDorsal-S and LvDorsal-L mRNA were detected in all examined tissues (**Figure 4B**). Compared with LvDorsal-S, LvDorsal-L was the major dorsal isoform in all tested tissues. LvDorsal-L was highly expressed in hemocytes, gills, and intestines. LvDorsal-S was relatively abundant in hemocytes and showed low expression levels in other tissues. Therefore, hemocytes were chosen to measure transcriptional changes in LvDorsal-S and LvDorsal-L after PAMP and pathogen infection. The results showed that from 4 to 72 hours post *V. parahaemolyticus* infection, LvDorsal-L plays a continuous role in the immune response induced by *V. parahaemolyticus* (**Supplementary Figure 4**).

AMPs play a vital role in protecting the host from invading microorganisms and are primarily induced by NF-κB. Dual-luciferase reporter assays were performed to determine the effect of LvDorsal-S and LvDorsal-L on the regulation of AMP induction. As shown in **Figure 4C**, LvDorsal-L significantly activated the promoters of AMP genes, including the Drosophila antimicrobial peptide DmDpt (~7.21-fold), DmDrs (~124.74-fold), DmAttA (~152.70-fold), Penaeus antimicrobial peptide Pm411 (~2.27-fold), Pm536 (~1.64-fold), LvCrustin1 (~245.49-fold), and artificial 4×NF-κB (~80.88-fold). Unexpectedly, overexpression of LvDorsal-S only slightly increased the promoter activities of DmAttA (~2.04-fold) and LvCrustin1 (~2.16-fold) and even decreased DmDpt (~0.61-fold) and Pm536 (~0.59-fold) promoter activity. The activation of the DmDrs, Pm411, or 4×NF-κB promoters did not change significantly during LvDorsal-S overexpression. LvCrustin1, also known as LvCrustina-2, belongs to type II crustin, which has antibacterial activity against both gram-positive and gram-negative bacteria and was the first crustin identified in *L. vannamei* (35). These results suggest that LvDorsal is involved in the regulation of shrimp and Drosophila antimicrobial peptides, while LvDorsal-L is the isoform with stronger inductive activity.

### 3.5 LvDorsal-L protected the host from *V. parahaemolyticus* infection

**Figure 5:**
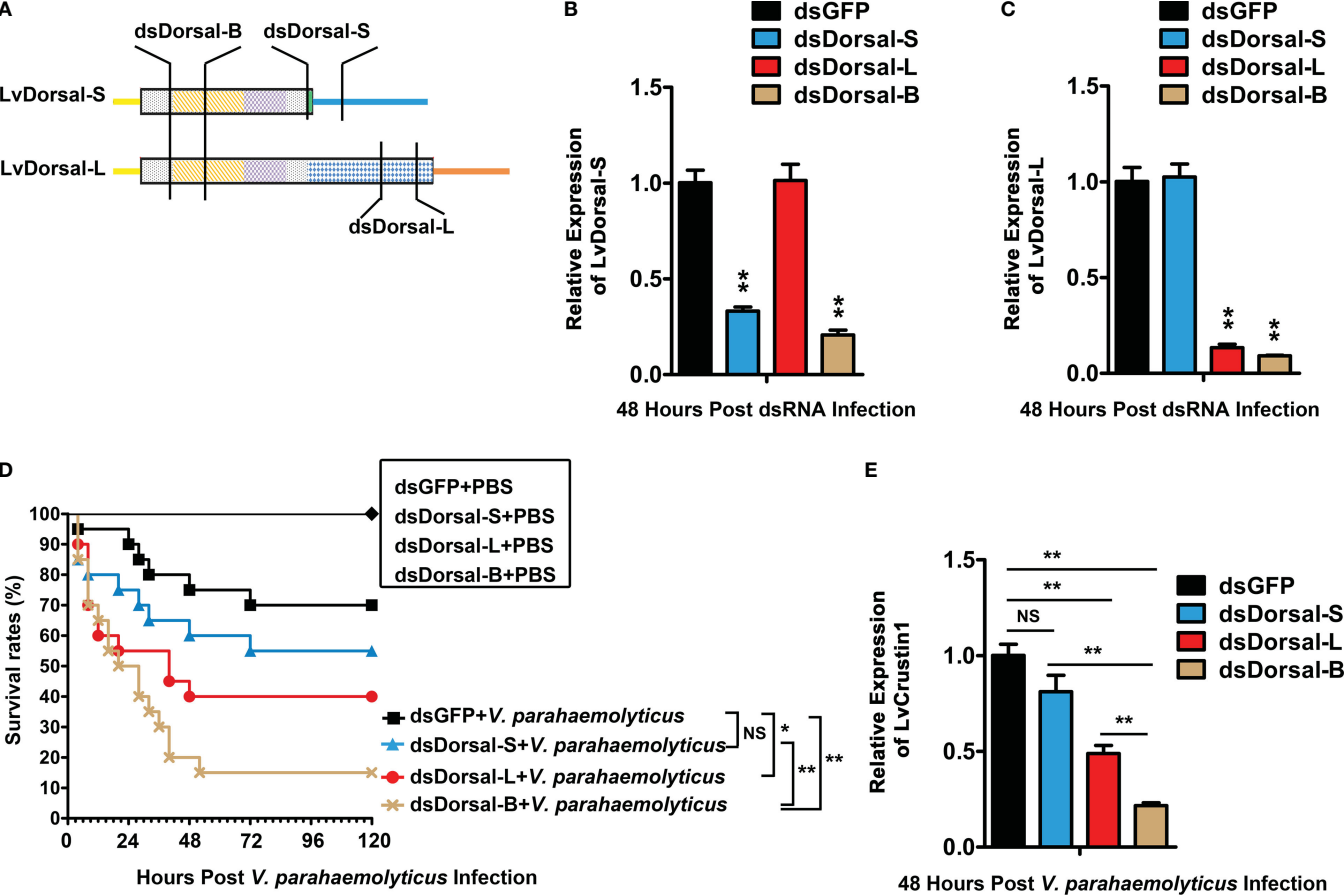
Function of LvDorsals during *V. parahemolyticus* infection. **(A)** Illustration of dsRNA-LvDorsal-S, dsRNA-LvDorsal-L, and dsRNA-LvDorsal-B target sequences. **(B)** QPCR analysis of the silencing efficiency of LvDorsal-S. **(C)** QPCR analysis of LvDorsal-L silencing efficiency. **(D)** Survival rates of LvDorsal-silenced shrimp during *V. parahemolyticus* infection. **(E)** QPCR analysis of LvCrustin1 expression levels after *V. parahemolyticus* infection in dsRNA-LvDorsal-S, dsRNA-LvDorsal-L, and dsRNA-LvDorsal-B groups. The hemocyte from 3 shrimp were sampled for QPCR. **p < 0.01; *p < 0.05; ns, no significance.

RNAi of LvDorsal-S, LvDorsal-L, and LvDorsal-B was used to investigate whether LvDorsal-S and LvDorsal-L were involved in host defense against *V. parahaemolyticus*. The dsRNA-LvDorsal-S and dsRNA-LvDorsal-L primers were situated at the C-terminus of LvDorsal-S and LvDorsal-L, respectively. In contrast, the dsRNA-Dorsal-B primers were situated in the RHD DNA-binding domain to knockdown both LvDorsal-S and LvDorsal-L, respectively (**Figure 5A**). As shown in **Figure 5B**, dsRNA-LvDorsal-S and dsRNA-LvDorsal-B remarkably suppressed the transcription of LvDorsal-S, while LvDorsal-L was inhibited by dsRNA-LvDorsal-L and dsRNA-LvDorsal-B (**Figure 5C**). The shrimp were then injected with *V. parahaemolyticus* 48 h post-dsRNA injection, and survival rates were monitored for 120 h after the infection. As shown in **Figure 5D**, the survival rates in the dsRNA-LvDorsal-L and dsRNA-LvDorsal-B groups were lower than those in the dsRNA-GFP control group, indicating that shrimp were more vulnerable to *V. parahaemolyticus* infection. Notably, the survival rate of the dsRNA-LvDorsal-S group was not significantly different (p = 0.28) but demonstrated a lower trend than the control group. To gain more information on the effect of LvDorsal on LvCrustin1 induction during *V. parahaemolyticus* infection, a parallel experiment was performed to explore the transcription levels of LvCrustin1 in LvDorsal-silenced shrimp following infection. In accordance with the survival rates, the transcription level of LvCrustin1 was much lower in the dsRNA-LvDorsal-L and dsRNA-LvDorsal-B groups than in the control group (**Figure 5E**). Overall, our data suggests that LvDorsal-L, rather than LvDorsal-S, is a key factor in protecting the host from *V. parahaemolyticus* infection and inducing LvCrustin1 production.

### 3.6 LvToll1/2/3/5/9 were involved in regulating Dorsal-L phosphorylation responses to *V. parahaemolyticus* infection

**Figure 6:**
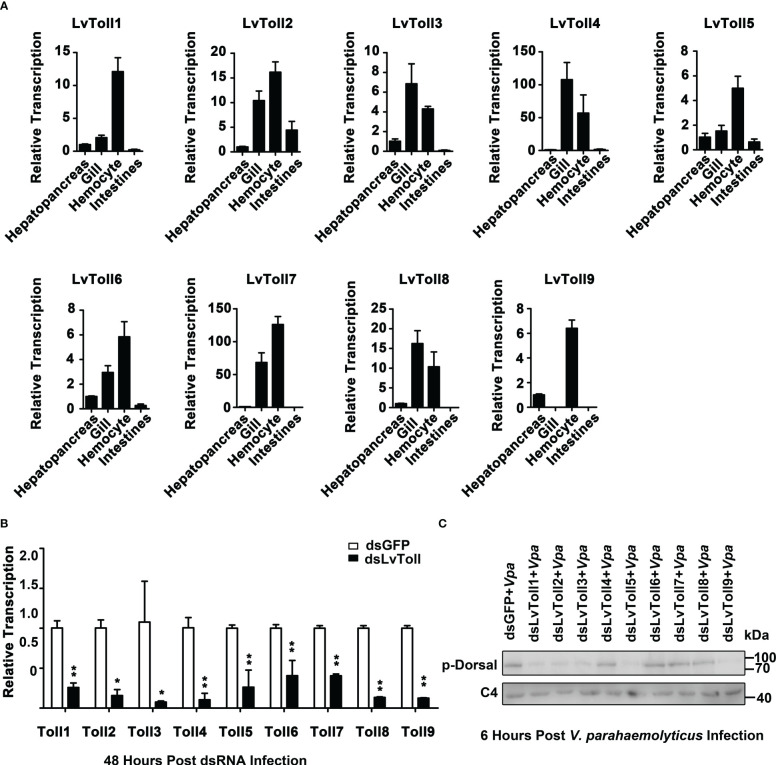
Roles of LvTolls in regulating LvDorsal-L during *V. parahemolyticus* infection. **(A)** Tissue distributions of LvToll1-LvToll9 were detected by qPCR. **(B)** QPCR analysis of the efficiency of dsRNA-LvTolls silencing. EF1-α gene was used as internal control. **(C)** The phosphorylation levels of LvDorsal-L after LvTolls knocked down during *V. parahemolyticus* infection. The hemocytes from 3 shrimp per group were sampled for QPCR. And hemocytes from nine shrimp per group were sampled for western blotting. *p < 0.05: **p < 0.01.

Given that LvDorsal-L was the predominant isoform that responded to *V. parahaemolyticus* infection in shrimp, we presumed that activation of LvDorsal-L was critical for shrimp action against *V. parahaemolyticus*. Considering that LvToll1/2/3/5/9 could bind to LPS, we wondered whether Toll1, Toll2, Toll3, Toll5, and Toll9 were involved in LvDorsal-L activation during *V. parahaemolyticus* infection. To select the proper organ for the following experiments, the tissue distribution of LvTolls was assessed using qPCR. The results indicated that all nine Tolls were highly expressed in hemocytes (**Figure 6A**), therefore, hemocytes were chosen as the target tissue in the evaluation of the silencing efficiencies for each Toll by qPCR 48 h post dsRNA injection (**Figure 6B**). Next, we challenged RNAi-treated shrimp with *V. parahaemolyticus* and subsequently analyzed the phosphorylation level of LvDorsal by western blotting. As shown in **Figure 6C**, the phosphorylation levels of LvDorsal were inhibited in the dsRNA-LvToll1, dsRNA-LvToll2, dsRNA-LvToll3, dsRNA-LvToll5, and dsRNA-LvToll9 groups, suggesting that LvToll1/2/3/5/9 may be involved in regulating LvDorsal-L activation during *V. parahaemolyticus* infection.

### 3.7 LvTRAF6-LvTAB2-LvTAK1 participated in Toll-regulated LvDorsal activation during *V. parahaemolyticus* infection

**Figure 7:**
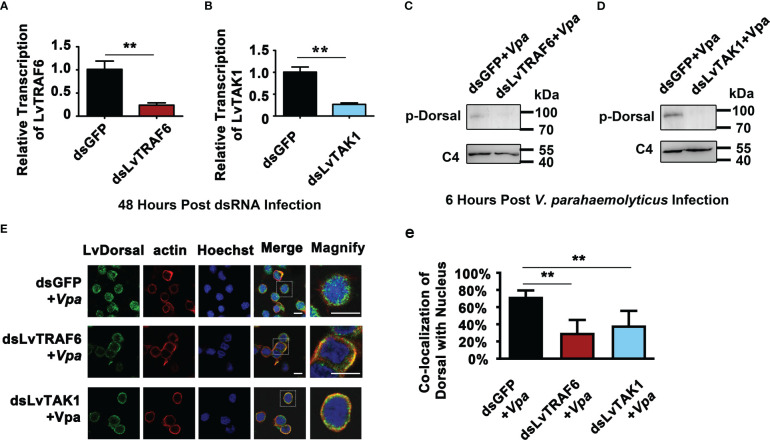
Roles of LvTRAF6-LvTAB2-LvTAK1 complex in regulating LvDorsal-L activation during *V. parahemolyticus* infection. **(A-B)** The RNA interference efficiencies of dsRNA-LvTRAF6 **(A)** and dsRNA-LvTAK1 **(B)** were examined by qPCR. The hemocytes from 3 shrimp per group were sampled for QPCR. **(C-D)** Phosphorylation of LvDorsal-L after dsRNA-LvTRAF6 **(C)** and dsRNA-LvTAK1 **(D)** injection following with *V. parahaemolyticus* infection were analyzed by western blotting with anti-p-LvDorsal antibody. Hemocytes from nine shrimp per group were sampled for western blotting. **(E)** LvDorsal subcellular location in hemocytes in response to dsRNA-LvTRAF6 and dsRNA-TAK1 during *V. parahaemolyticus* infection. The scale bar = 5 μm. **(e)** Co-localization of LvDorsal and Hochest-stained nucleus in hemocytes was calculated by WCIF ImageJ software and analyzed statistically by student’s T test (**p < 0.01). Hemocytes from three shrimp per group were sampled for immunofluorescence.

Although we indicated that LvToll1/2/3/5/9 activated LvDorsal-L, it was still unclear whether the TRAF6-TAB2-TAK1 complex, a critical component of the TLR pathway, stimulated LvDorsal-L activity. In this study, RNAi was used to knock down LvTRAF6 or LvTAK1 in shrimp, followed by *infection with V. parahaemolyticus*. As shown in **Figure 7A**, the transcription of LvTRAF6 was successfully inhibited by dsRNA-LvTRAF6, 48 h post dsRNA injection. Efficient silencing of LvTAK1 was observed in the dsRNA-LvTAK1 group after 48 h post-dsRNA injection using qPCR (**Figure 7B**). Six hours post-*V. parahaemolyticus* infection, the phosphorylation of LvDorsal-L in hemocytes was much lower in the dsRNA-LvTRAF6 and dsRNA-LvTAK1 groups than in the dsRNA-GFP group (**Figures 7C, D**). In accordance with the Dorsal-L phosphorylation results, immunofluorescence experiments in the hemocytes demonstrated that endogenous LvDorsal-L (green fluorescence) co-localized less with the nucleus after 6 h post-infection in both dsRNA-LvTRAF6 and dsRNA-LvTAK1 hemocytes, compared to dsRNA-GFP hemocytes (**Figures 7E**). These findings strongly suggested that the TRAF6-TAB2-TAK1 complex in shrimp is essential for LvDorsal-L activation during *V. parahaemolyticus* infection.

**Figure 8:**
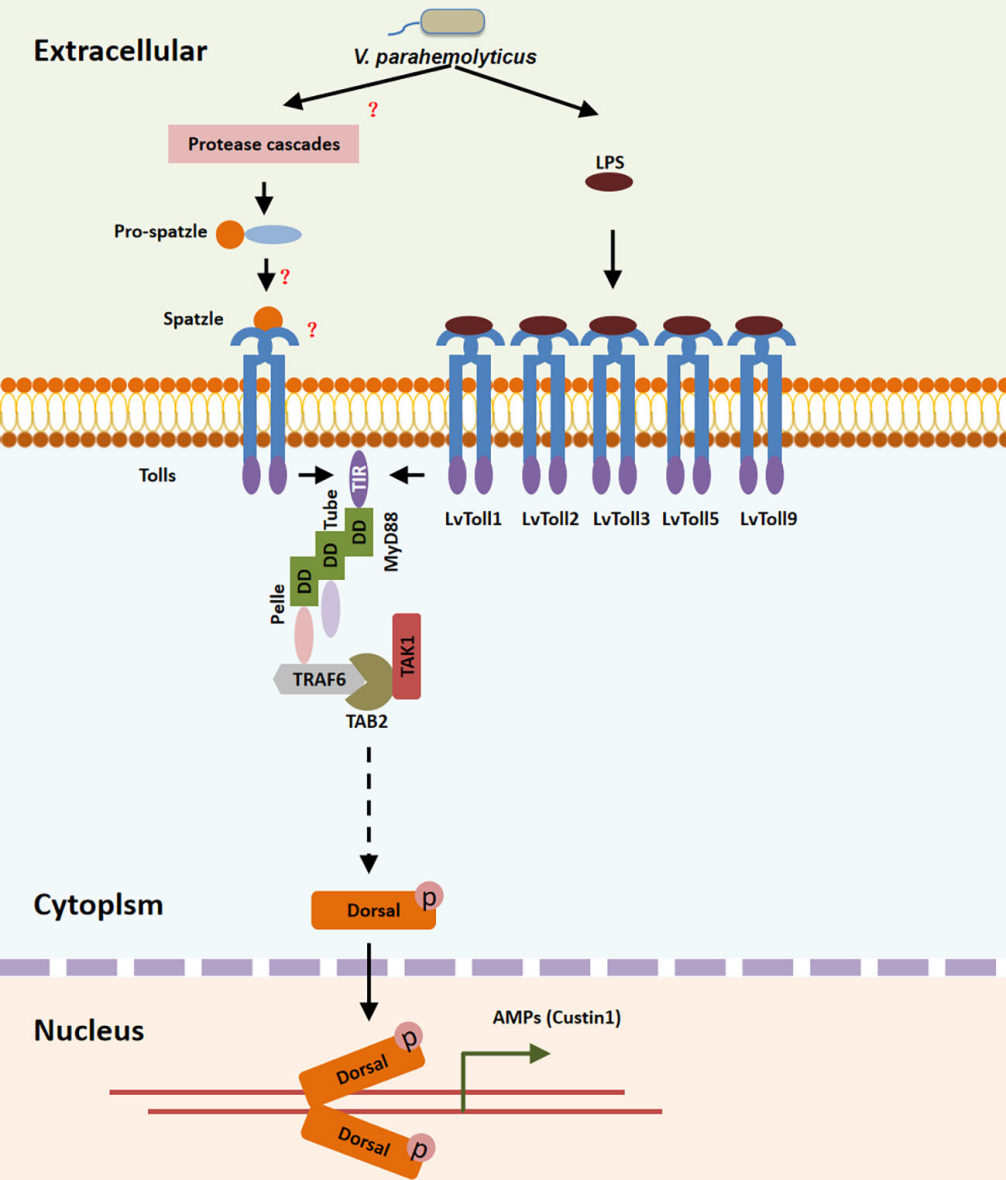
Toll/MyD88/Tube/Pelle/TRAF6-TAB2-TAK1 pathway regulates the LvDorsal-L induced anti-bacterial function. During *V. parahaemolyticus* infection, LPS was recognized by the extra-cellular domain of LvToll1, LvToll2, LvToll3, LvToll5 and LvToll9, the TIR of which interacted with MyD88 and induced MyD88-Tube-Pelle-TRAF6 signaling. LvTAB2 bridged TRAF6 to TAK1, allowing LvDorsal activation and LvCrustin1 induction.

In summary, during *V. parahaemolyticus* infection, LvToll1/2/3/5/9 recognized LPS and recruited the TRAF6-TAB2-TAK1 complex *via* the MyD88-Tube-Pelle cascade, leading to the activation of LvDorsal-L, which plays a major antibacterial role in *L. vannamei via* the induction of LvCrustin1, implying that shrimp may fight bacterial infection *via* the Toll-TRAF6/TAB2/TAK1-Dorsal pathway (**Figure 8**).

## 4 Discussion

One of the most intriguing questions in immunology is how the host recognizes pathogens and triggers immune pathways. To cope with varying types of infection, different immune recognition systems have evolved in different species. The Toll/TIR-NF-κB pathway is one of the most important immune pathways in both vertebrates and invertebrates and was first identified in Drosophila (34, 36). Over hundreds of millions of years, approximately 30 invertebrate phyla have diverged along distinct evolutionary trajectories, including those in marine, freshwater, and terrestrial environments (37). Therefore, despite significant immunological similarities among invertebrates, their immune systems are non-homogeneous, complex, and broad-spectrum responsive.

The invertebrate immune system is non-homogeneous. The innate immunity recognition is based on the detection of the composition and conserved products of microbial metabolism. LPS is produced by bacteria (38), thus LPS can be seen as the microbial invader’s molecular signature, which may be recognized by germ-line-encoded receptors, such as TLR or peptidoglycan recognition proteins. From the standpoint of PAMP recognition, shrimp Tolls have more similarities with TLRs than with insect Tolls. Unlike Drosophila Tolls, which cannot directly recognize PAMP, we discovered that LvToll1, LvToll2, LvToll3, LvToll5, and LvToll9 can respond to bacterial infection by binding to LPS. It should be noted that Tolls in shrimp do not correspond to homologs of TLRs or Tolls found in mammals or Drosophila. Instead, Tolls in *L. vannamei* were designated as LvToll1 to LvToll9 according to the time order of their cloning. There have been reports that Toll can bind directly to bacteria as well as LPS and peptidoglycan (PGN) in other aquatic invertebrate species such as *Marsupenaeus japonicus* (39), *Crassostrea gigas* (40), and *Hyriopsis cumingii* (41). It is still unknown why LvToll1, 2, 3, 5, and 9 could recognize LPS rather than other Tolls. We hypothesized that this was due to differences in the extracellular domain structure of these Tolls. The numbers of extracellular LRRs in these nine Tolls varied greatly (31), suggesting that these Tolls can respond to a variety of PAMPs. In terms of signal transduction, given that dTRAF2 is insignificant in response to bacterial infection (22), but LvTRAF6 responds to bacterial infection and induction of AMPs (42), we were not surprised to find a TRAF6-TAK1-TAB2 complex in shrimp, which has not yet been found in Drosophila. *In vitro* immunoprecipitation tests revealed that TAK1 and TAB2 could interact *via* the CC domain which is similar to that which has been observed in mammals (43). In mammals, TAB2 binds to TRAF6 *via* the C-terminal ZnF domain, whereas no fragment of TRAF6 alone can interact with TAB2 (43). The MATH domain of LvTRAF6 is sufficient to connect with LvTAB2 in shrimp, however, the entire length of LvTAB2 is necessary for the LvTAB2-LvTRAF6 interaction. While the C-terminal of TAB2 in mammals forms dimeric complexes with TAK1 and TRAF6, the TAK1-TAB2-TRAF6 complex requires a full-length TAB2 in both shrimp and mammals.

The invertebrate immune system is complex. It is regulated not only by activating or suppressing factors but also by different isoforms of the same gene. Many components of the TLR/NF-κB pathway that have different isoform lengths with different or opposite functions have been reported in invertebrates (27, 44–46). For example, in *Anopheles gambiae* mosquitos, REL2-F is involved in the defense against gram-positive *S. aureus* bacteria, whereas REL2-S participated in the defense against gram-negative *Escherichia coli* bacteria (44). Only the short Rel2-S isoform of Rel2 confers protection against *Plasmodium falciparum*, not the long Rel2-F isoform (45). In shrimp, MyD88 and Tube each have two variants. LvMyD88-1 lacks the Box1 region, but has higher activity in regulating AMPs compared to LvMyD88 (46). Both LvMyD88 and LvMyD88-1 have been shown to interact with LvTube/LvTube-1 and LvTube-1, with a stronger activation effect on arthropod AMP promoters (27). In this study, we further identified LvDorsal-L, a new LvDorsal isoform that is much larger than LvDorsal-S at the C-terminus. Evidence from tissue distribution, immune response, AMP regulation, and bacterial infection mortality after RNAi suggest that LvDorsal-L is not only the major Dorsal isoform, however, it does play a predominant role in preventing shrimp from *V. parahaemolyticus* infection by inducing AMPs. Shrimps can selectively use these isoforms to produce different signal transduction levels, eliciting varying degrees of responses in different tissues against different pathogen invasions.

The invertebrate immune system is broad-spectrum responsive, particularly in aquatic invertebrate. The habitats of aquatic invertebrates are typically characterized by bacteria and viruses. *V. parahaemolyticus*, a gram-negative bacterium, is a dominant autochthonous microflora found in estuarine and coastal marine environments and is linked to aquatic animal diseases (47). Interestingly, in mice, only one of the 12 TLR members, TLR4, recognizes LPS (48) whereas in shrimp, five of the nine Tolls have such a function. Therefore, aquatic invertebrates have evolved immune mechanisms that allow multiple receptors to recognize LPS and initiate an immune response. Our results also demonstrate how shrimp induce AMPs rapidly and widely in response to *V. parahaemolyticus* infection. In invertebrates and vertebrates, TAK1 is required for the activation of the NF-κB and MAPK signaling pathways, as it is the kinase upstream of IKK and MAPK kinase4 (49, 50). Recently, LvTAK1 was shown to possess antibacterial activity by mediating the activation of MAPK and Relish pathways (30). In this study, TAK1 is shown to be critical for Dorsal activation in invertebrates. As a result of *V. parahaemolyticus* infection, lipopolysaccharides on the outer cell envelope of gram-negative bacteria interact with Toll1, Toll2, Toll3, Toll4, Toll5, and Toll9, thereby activating Toll-MyD88-Tube-Pelle-TRAF6/TAB2/TAK1 signaling. Activated TAK1 boosts AMP induction *via* the MAPK, Relish, and Dorsal pathways to eradicate invading bacteria.

In summary, our results indicated that among all Toll receptors, LvToll1/2/3/5/9 recognize *V. parahaemolyticus* by interacting with LPS, thereby recruiting the LvTRAF6-LvTAB2-LvTAK1 complex and inducing activation of LvDorsal-L, the predominant dorsal isoform in *L. vannamei*, which plays an important role in protecting shrimp from *V. parahaemolyticus* infection *via* LvCrustin1 induction (**Figure 8**).

## Data availability statement

The original contributions presented in the study are included in the article/**Supplementary Material**. Further inquiries can be directed to the corresponding authors.

## Ethics statement

The animal study was reviewed and approved by the Institutional Animal Care and Use Committee (IACUC), Sun Yat-Sen University. Written informed consent was obtained from the owners for the participation of their animals in this study.

## Author contributions

CL, JH, and SW conceived and designed the experiments. SW, HL, QL, SL, and BY performed the experiments and analyzed data. SW and CL wrote the draft manuscript. SW, HL, CL, and JH acquired finding. CL was responsible for forming the hypothesis, project development and submitting the manuscript. All authors contributed to the article and approved the submitted version.
