# Supplementary material for: Signaling events induced by lipopolysaccharide-activated Toll in response to bacterial infection in shrimp
Source: Front Immunol. 2023 Feb 3;14:1119879. doi: 10.3389/fimmu.2023.1119879 (PMC9936618; doi:10.3389/fimmu.2023.1119879)
Supplement: Supplementary file 1 [file DataSheet_1.docx]

**Supplemental Figures**

**Supplemental Figure 1.**


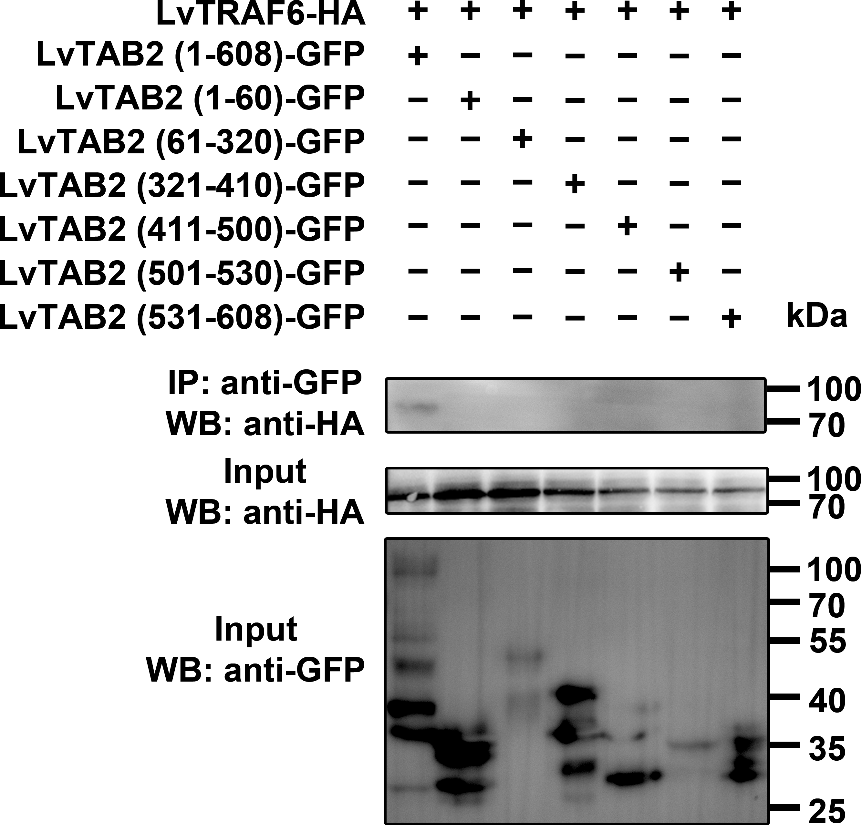


**Supplemental Figure 1. LvTAB2 and LvTRAF6 interaction required the entire length of LvTAB2.** HA-tagged LvTRAF6 could only combine with full length of GFP-tagged LvTAB2.

**Supplemental Figure 2.**


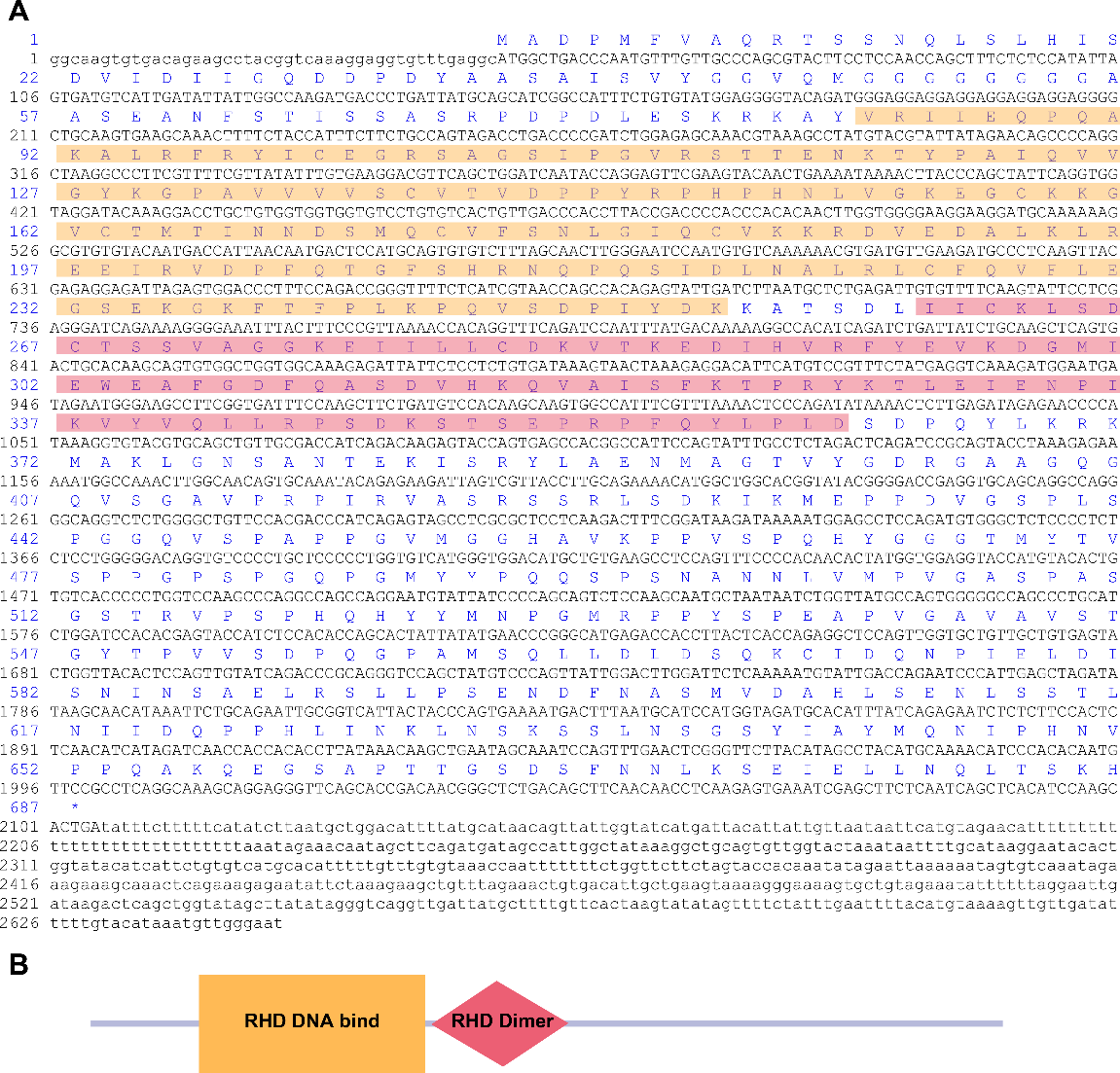


**Supplemental Figure 2. Sequence of LvDorsal-L.** Sequences of LvDorsal-L were obtained from NCBI. Specific primers were designed to amplify the nucleotide sequences from L. vannamei to confirm the sequences. The obtained PCR products were inserted into a pEASY-T1 Cloning Vector (TransGen Biotech, China) and identified by sequencing. The protein domains of LvDorsal-L were analyzed using SMART (Simple Modular Architecture Research Tool, http://smart.embl.de/). (A) The full-length cDNA sequence and deduced amino acid sequences of LvDorsal-L. Nucleotides and amino acids were numbered on the left of the sequences. Amino acid sequences were represented with one-letter codes above the nucleotide sequence. LvDorsal-L ORF nucleotide sequence was shown in uppercase letters, while the 5' and 3'-UTR sequences were shown in lowercase. The RHD DNA binding domain was shaded in orange, while the RHA dimer domain was shaded in red. (B) The structure of LvDorsal-L protein.

**Supplemental Figure 3.**


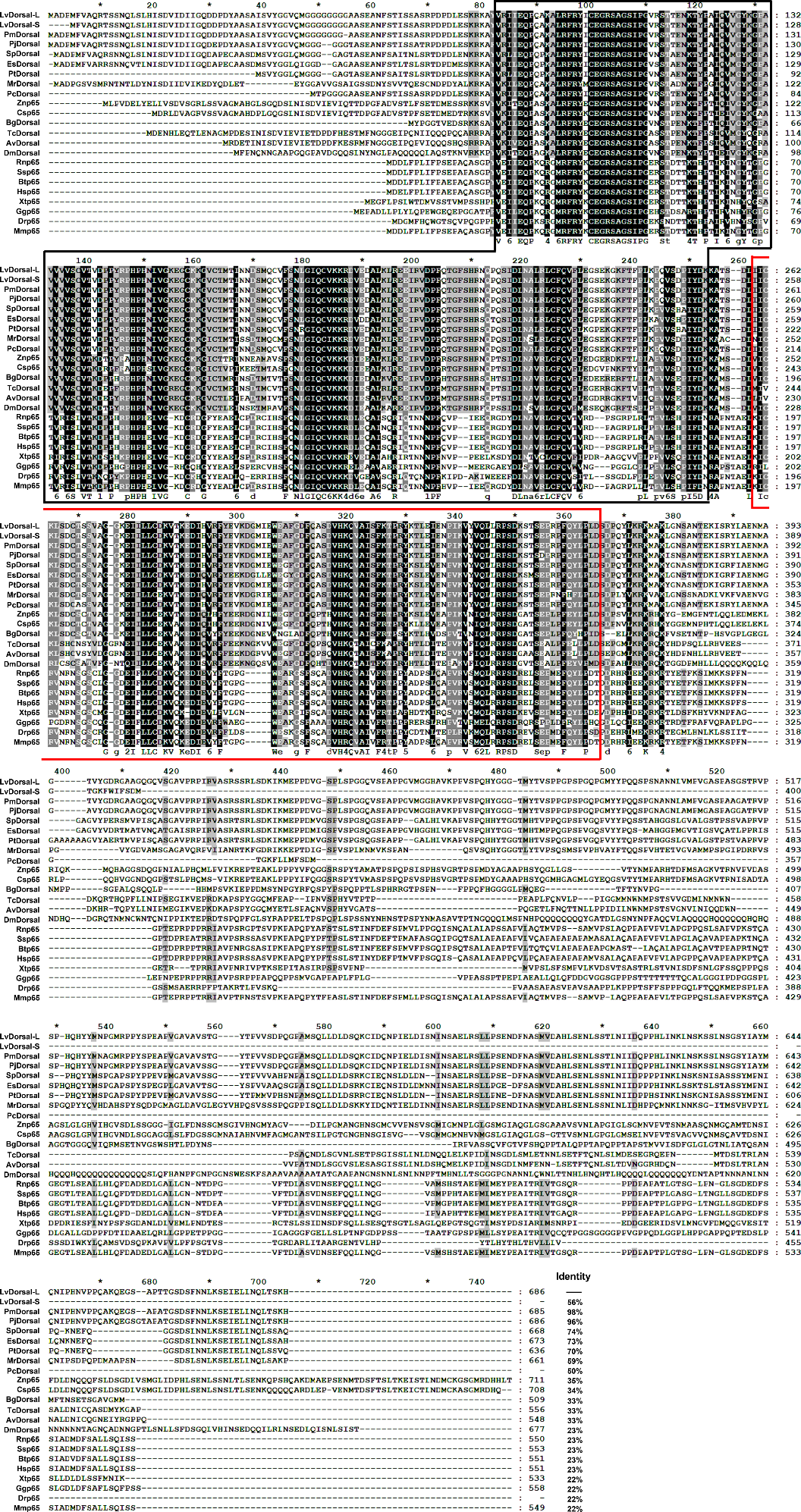


**Supplemental Figure 3. Multiple sequence alignment of the Dorsal proteins.** The alignment of Dorsal homologs was retrieved by using BLAST and Clustal X v2.0 program. The methods for the similarity measurement were described previously using GeneDoc. The identical amino acid residues were shaded in black, while the similar residues were shaded in gray. The RHD DNA binding domain was boxed with black lines, and the RHA dimer domain was boxed with red lines. On the right, the amino acid identities of LvDorsal-L and other Dorsals are shown. Proteins analyzed are listed below: *Litopenaeus vannamei* Dorsal-L (ROT84343.1); *Penaeus monodon* Dorsal (AYF59251.1); *Portunus trituberculatus* Dorsal (AXB88326.1); *Penaeus japonicus* Dorsal (AME17867.1); *Penaeus chinensis* Dorsal (ACJ36225.1); *Eriocheir sinensis* Dorsal (AHG95994.1); *Scylla paramamosain* Dorsal (QHA94739.1); *Macrobrachium rosenbergii* Dorsal (ANH22235.1); *Zootermopsis nevadensis* p65 (XP_021925458.1); *Cryptotermes secundus* p65 (XP_023722233.1); *Blattella germanica* Dorsal (CEG62431.1); *Tribolium castaneum* Dorsal (NP_001034507.1); *Asbolus verrucosus* Dorsal (RZB54393.1); *Drosophila melanogaster* Dorsal (NP_001163000.1); *Mus musculus* p65 (AAA39811.1); *Xenopus tropicalis* p65 (NP_001001211.1); *Gallus gallus* p65 (NP_990460.1); *Rattus norvegicus* p65 (NP_954888.1); *Sus scrofa* p65 (NP_001107753.1); *Bos taurus* p65 (NP_001073711.1); *Danio rerio* p65 (NP_001001839.2); *Homo sapiens* p65 (NP_068810.3); *Litopenaeus vannamei* Dorsal-S (ACZ98167.1).

**Supplemental Figure 4.**


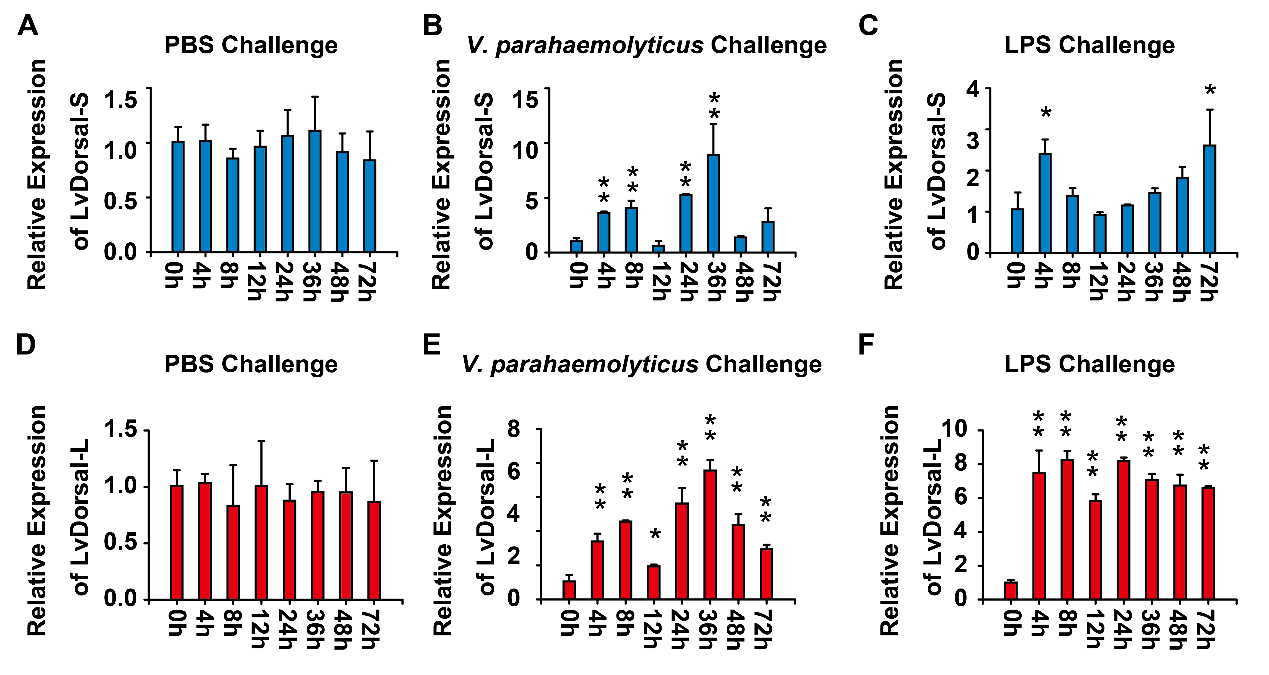


**Supplemental Figure 4. Transcription profiles of LvDorsals in hemocytes from LPS or *V. parahaemolyticus* challenged *L. vannamei*.** Transcription profiles of LvDorsal-S in hemocytes from PBS (A), *V. parahaemolyticus* (B) and LPS (C), as well as transcription profiles of LvDorsal-L in hemocytes from PBS (D), *V. parahaemolyticus* (E) and LPS (F) were analyzed by qPCR. The transcription level detected at 0 hours post injection of each group was set as 1.0. The transcription values were normalized to those of EF-1α, and the data were presented as the means ± SD of triplicate assays (**: *p* < 0.01; *: *p* < 0.05).
